# The Effect of Exercise on Pain in People with Cancer: A Systematic Review with Meta-analysis

**DOI:** 10.1007/s40279-023-01862-9

**Published:** 2023-05-22

**Authors:** Melanie Louise Plinsinga, Ben Singh, Grace Laura Rose, Briana Clifford, Tom George Bailey, Rosalind Renee Spence, Jemma Turner, Michel Willem Coppieters, Alexandra Leigh McCarthy, Sandra Christine Hayes

**Affiliations:** 1 School of Health Sciences and Social Work, Menzies Health Institute Queensland, Griffith University, Brisbane and Gold Coast, Australia; 2 Allied Health and Human Performance, Alliance for Research in Exercise, Nutrition and Activity, University of South Australia, Adelaide, Australia; 3 School of Nursing, Midwifery and Social Work, The University of Queensland, and Mater Research Institute, Brisbane, Australia; 4 School of Health Sciences, University of New South Wales, Sydney, Australia; 5 Faculty of Behavioural and Movement Sciences, Amsterdam Movement Sciences-Musculoskeletal Health Program, Vrije Universiteit Amsterdam, Amsterdam, The Netherlands

## Abstract

**Introduction:**

Cancer-related pain is common and undertreated. Exercise is known to have a pain-relieving effect in non-cancer pain.

**Objectives:**

This systematic review aimed to evaluate (1) the effect of exercise on cancer-related pain in all cancers, and (2) whether the effect of exercise differed according to exercise mode, degree of supervision, intervention duration and timing (during or after cancer treatment), pain types, measurement tool and cancer type.

**Methods:**

Electronic searches were undertaken in six databases to identify exercise studies evaluating pain in people with cancer, published prior to 11 January 2023. All stages of screening and data extraction were conducted independently by two authors. The Cochrane risk of bias tool for randomised trials (RoB 2) was used and overall strength of evidence was assessed using the GRADE approach. Meta-analyses were performed overall and by study design, exercise intervention and pain characteristics.

**Results:**

In total, 71 studies reported in 74 papers were eligible for inclusion. The overall meta-analysis included 5877 participants and showed reductions in pain favouring exercise (standardised mean difference − 0.45; 95% confidence interval − 0.62, − 0.28). For most (> 82%) of the subgroup analyses, the direction of effect favoured exercise compared with usual care, with effect sizes ranging from small to large (median effect size − 0.35; range − 0.03 to − 1.17). The overall strength of evidence for the effect of exercise on cancer-related pain was very low.

**Conclusion:**

The findings provide support that exercise participation does not worsen cancer-related pain and that it may be beneficial. Better pain categorisation and inclusion of more diverse cancer populations in future research would improve understanding of the extent of benefit and to whom.

**PROSPERO registration number:**

CRD42021266826.

**Supplementary Information:**

The online version contains supplementary material available at 10.1007/s40279-023-01862-9.

## Key Points

**Table:** 

Evidence consistently favours participation in exercise for cancer-related pain, although quality was graded as very low.
Exercise participation does not worsen cancer-related pain and may be beneficial in pain management.

## Introduction

Cancer-related pain is one of the most common and debilitating cancer-related side effects across all cancers [1, 2], and its prevalence is expected to rise alongside the projected increase in cancer survivors in the coming decades [3, 4]. The term cancer-related pain pertains to pain of any origin and may present at any point along the cancer continuum (diagnosis through to end of life). Cancer-related pain can include but is not limited to visceral pain, neuropathic pain, nociplastic pain, nociceptive pain, bone and musculoskeletal pain [4]. These types of pain may originate from the tumour, from cancer treatment (i.e. surgery, chemotherapy, radiotherapy) or from survivorship conditions (i.e. lymphoedema) [4]. Further, cancer-related pain can vary in duration (acute, subacute, chronic), site (i.e. shoulder, stomach, feet/hands), intensity and timing of presentation (before, during and after treatment) [1]. For the purpose of this review, we refer to cancer-related pain as any type of pain of any cause presenting along the cancer continuum.

Cancer-related pain has a profound impact on daily activities (including personal, social and occupational roles), mental health and overall quality of life [5–7]. Population-based data involving 4526 cancer patients (including all stages of disease at diagnosis, and during and after treatment) indicate that 35% of cancer survivors experienced pain on most days of the week within the previous 6 months [8]. Further, findings from a meta-analysis (117 studies, *n* = 63,533) showed that overall, 38% of all cancer patients reported moderate-to-severe pain, with a prevalence of 39% after curative treatment, 55% during treatment and 66% in people with advanced, metastatic or terminal disease [1].

Effective relief of cancer-related pain depends on multiple factors including pain causation, type, duration and intensity. The most common pain management option is the intake of analgesics, with non-steroidal anti-inflammatory drugs and/or opioids endorsed by international pain societies [9, 10] and the World Health Organization [11]. However, pharmacological treatment is not effective for some types of pain. It is also associated with a risk of adverse physical and psychological health-related problems which, when present, require additional management [12, 13]. Non-pharmacological pain management strategies, including pain educational programmes to reduce patient-related barriers and to improve knowledge and communication with healthcare professionals [14, 15], and psychosocial interventions (involving relaxation techniques, cognitive–behavioural therapy, music therapy, mindfulness- and acceptance-based interventions, and supportive-expressive group therapy) have also been explored [16]. However, the heterogeneity across the interventions evaluated to date has contributed to an inconsistent evidence base overall for these therapies [14–16].

Exercise interventions of mixed mode and intensity are reported as beneficial to reduce pain in non-cancer populations, including pain due to osteoarthritis and fibromyalgia [17, 18]. Within the cancer population, observational evidence suggests that people participating in more physical activity report less pain than people who engage in less physical activity [19]. Previous systematic reviews have synthesised evidence derived from testing physiotherapy-based exercise interventions targeting shoulder pain following breast cancer, and exercise interventions aimed at preventing chemotherapy-induced peripheral neuropathies, with evidence supporting benefit [20, 21]. However, to date no systematic reviews with meta-analyses have been undertaken to quantify the potential effect of exercise on cancer-related pain. There is also limited exploration, and therefore understanding, as to whether exercise, pain or patient characteristics influence any potential exercise effect.

The purpose of this systematic review and meta-analysis was therefore to evaluate (1) the effects of exercise on cancer-related pain in all cancers, and (2) whether the effect of exercise differed according to exercise mode, degree of supervision and intervention duration and timing (during or after cancer treatment), pain types and measurement tool, and cancer type.

## Methods

The protocol for this systematic review was prospectively registered on PROSPERO (CRD42021266826).

### Search Strategy and Selection Criteria

Electronic searches were undertaken in the following databases: Cochrane Library, PubMed, CINAHL, SPORTDiscus (via EBSCOhost), EMBASE and Scopus for studies published up to 10 January 2023. The search strategy was developed with assistance from an institutional librarian and involved a combination of free-text words and subject heading terms for ‘pain’, ‘exercise’ and ‘physical activity’. The full search strategy per database can be found in Table S1. Supplementary searches of reference lists of included studies were undertaken.

The selection criteria were developed using the Participant, Intervention, Comparator and Outcome (PICO) framework and can be found in Table 1 [22]. Articles that were not published in English were excluded.

**Table 1:** Selection criteria

P—Participants	Humans > 18 years old who were diagnosed with any type of cancer and at any stage of treatment (either awaiting, undergoing or completed any form of cancer treatment)
I—Interventions	Randomised controlled/clinical trials (RCTs) designed to evaluate exercise interventions. An RCT evaluating an exercise intervention was defined as a trial designed to evaluate exercise safety, feasibility or effectiveness with randomisation of participants. Exercise was considered as any form of planned, structured, and repetitive bodily movement performed to improve or maintain fitness, performance or health. Studies that involved exercise in addition to other interventions (e.g., dietary intervention, drug intervention) were excluded if the effects of exercise could not be isolated. Trials were eligible for inclusion irrespective of degree of intervention supervision, intervention length or exercise dosage prescribed. Interventions conducted at any time before, during or following treatment were eligible
C—Comparator	RCTs that involved a non-exercise control or usual care group were eligible. Non-randomised and single-group pre-post intervention studies (with no comparison group) were ineligible
O—Outcomes	The outcome of interest was pain. Any pain outcome assessed using any pain instrument or pain item/subscale on a non-pain instrument (e.g., pain subscale in a quality of life questionnaire) was eligible. Outcome data had to be assessed at baseline (pre-intervention) and post-intervention to be included

### Screening and Data Extraction

Using the selection criteria described above, a two-step screening process was conducted using Covidence (Veritas Health Innovation, Melbourne, Australia). The titles and abstracts of all identified studies were screened by two reviewers from a pool of six reviewers (MP, BS, TB, BC, GR, CA). Additional reviewers (SH, ALM) were consulted to resolve disagreements if necessary. Full texts were then retrieved, and the same process was followed for screening of full-text articles. Reference lists of included studies were also screened to identify potentially eligible studies.

For eligible studies, two independent reviewers (from a pool of six reviewers: MP, BS, GR, BC, RS, JT) extracted data regarding study characteristics, exercise details, participant characteristics (including cancer type and timing of exercise intervention with respect to cancer treatment) and pain outcomes in tabular format using pre-defined data fields in Microsoft Excel (Microsoft Corporation, 2018). Any pain data pre- and post-exercise intervention, as assessed via any method, were extracted. Exercise details that were extracted included exercise mode, intensity, duration of intervention, degree of supervision and timing of intervention with respect to timing of cancer treatment. Authors were contacted via email to maximise data extraction when needed.

### Risk of Bias

Risk of bias was assessed independently by two authors (from a pool of six authors: MP, BS, GR, BC, RS, JT) using version 2 of the Cochrane risk of bias tool for randomised trials (RoB 2) [23]. Disagreements were resolved by senior author SH. The RoB 2 is structured into a fixed set of domains of bias, focusing on different aspects of trial design, conduct and reporting. Articles were rated as having a ‘low risk of bias’, ‘high risk of bias’ or having ‘some concerns’ [23].

### Statistical Analyses

Review Manager (RevMan v5.4) was used to perform meta-analyses for all pain outcomes by comparing post-intervention means and standard deviations for the exercise and non-exercise/usual care groups of all randomised controlled trials (RCTs). Standardised mean differences (SMDs) with 95% confidence intervals (95% CIs) were calculated using generic inverse variance methods in a random-effects model for continuous data. In studies using more than one method to assess pain, extraction of data from Visual Analogue Scales and Numeric Rating Scales were prioritised over subscales from health-related questionnaires (such as SF-36).

The following planned subgroup analyses were performed to assess the potential effect of exercise on pain to be modified by: (1) exercise mode: aerobic, resistance, mixed mode (i.e. aerobic and resistance), yoga and other exercise (i.e. martial arts and rock climbing); (2) intervention duration: < 12 weeks or ≥ 12 weeks; (3) degree of intervention supervision: supervised (i.e. half or more than half of the exercise sessions were supervised face to face) or unsupervised (i.e. less than half of the exercise sessions involved face-to-face supervision; telehealth sessions were considered unsupervised); (4) pain types as defined by the individual studies (i.e. musculoskeletal pain, neuropathic pain); (5) pain measurement tools (i.e. European Organization for the Research and Treatment of Cancer Quality of Life Questionnaire (EORTC QLQ C30)—Pain Scale, SF-36—Bodily Pain Scale, Numeric (Pain) Rating Scale, Visual Analogue Scale, Neuropathic Pain Scale, Brief Pain Inventory, Western Ontario and McMaster Uni OA Index); (6) cancer type (i.e. breast, lung, colon and colorectal, head and neck, prostate and two or more cancer sites); and (7) intervention timing with respect to timing of cancer treatment (during and/or after chemotherapy and/or radiotherapy).

Sensitivity analyses were undertaken by assessing the effect of study design characteristics on the results: (1) sample size: *n* < 20, *n* = 21–59, *n* ≥ 60; (2) risk of bias: high risk of bias, some concerns or low risk of bias, as rated with the RoB 2; and (3) pain selection criterion: pain as inclusion criterion, pain as exclusion criterion (i.e. pre-existing or excessive pain states) or pain not listed as selection criterion.

The *I*^2^ statistic was assessed for statistical heterogeneity, with values above 30%, 50% and 75% considered moderate, substantial and considerable heterogeneity, respectively [24]. An effect size of 0.2 was considered small, 0.5 moderate and 0.8 large [25]. A *p* value of less than 0.05 was considered statistically significant.

### Overall Strength of the Evidence Pool

The overall strength of evidence was assessed with the Recommendation, Assessment, Development and Evaluation (GRADE) tool [26]. The GRADE domains included study design, heterogeneity, risk of bias, indirectness, imprecision and publication bias, and were rated as ‘not serious’, ‘serious’ and ‘very serious’ limitations as per the Cochrane recommendations [26]. Limitations on study design were considered not serious as all included studies were RCTs. Risk of bias (assessed with the RoB 2) limitations were considered not serious if evidence was mostly (> 50%) from studies with a low risk of bias, serious if evidence was mostly from studies of some risk of bias and very serious if evidence was mostly from studies of high risk of bias [26]. Inconsistency/heterogeneity limitations were considered not serious if *I*^2^ < 50%, serious if *I*^2^ was between 51 and 69%, and very serious if *I*^2^ ≥ 70% [26]. Indirectness was considered serious if evidence was mostly from studies of unclear risk of indirectness and very serious if evidence was mostly from studies with high risk of indirectness of evidence based on the GRADE directness items. Imprecision limitations were considered serious if data from < 400 participants were available per outcome and if the confidence interval around the SMD exceeded 0.5, and very serious if the confidence interval around the SMD exceeded 1.0. Publication bias was assessed by examining whether studies reported different results based on the number of included participants.

The strength of evidence was considered high to begin with, because all included studies were RCTs. Outcomes were downgraded based on the number of serious or very serious limitations on the GRADE items. The overall strength of evidence was categorised as high, moderate, low or very low.

## Results

### Literature Search

**Fig. 1:**
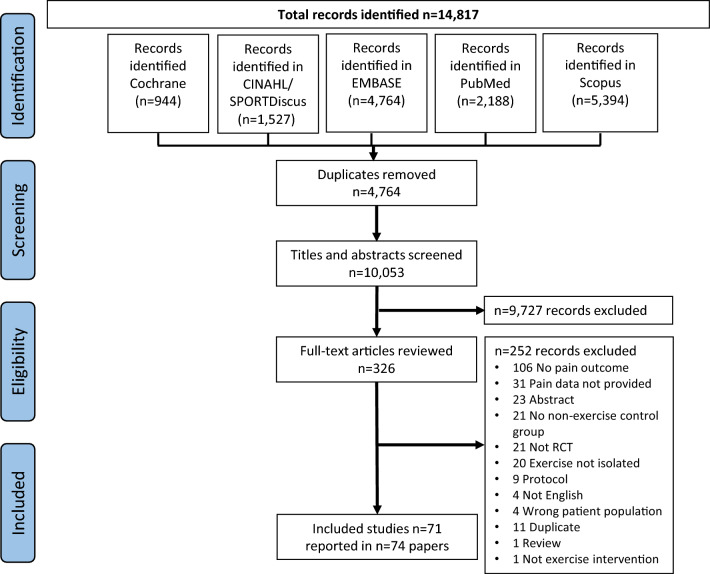
PRISMA flow diagram

A total of 14,817 articles were identified (Fig. 1). After removal of duplicates, 10,053 titles and abstracts were screened, and 326 full-text articles were retrieved. Additional information and/or pain data required for the meta-analysis were requested for 42 of the 326 articles that were considered for full-text review. Additional data required for 12 manuscripts were provided [27–37], while data for the remaining 30 articles were not. For three out of these 30 articles, means (SD) were estimated via web-based graph digitisers [38, 39] and through reported change scores from baseline to follow-up [40]. The remaining 27 articles were excluded from the review (Fig. 1; papers were added to numbers with reason for exclusion ‘pain data not provided’ at full-text review stage), the main reason being that authors used tools that included a pain subscale, but only reported the findings for the overall scale, not the pain subscale. In total, data from 71 studies reported in 74 papers were eligible for inclusion [27–100]. Five studies reported in eight papers included two exercise arms [31, 62, 71, 75, 76, 83, 87, 94].

### Participant and Intervention Characteristics

Study characteristics are presented in Table 2. The 71 RCTs included a total of 5877 people with cancer, with a mean (SD) age of 57 (10) years. Sample size per study ranged from 17 [70] to 287 [33]. Breast cancer was the most prevalent cancer type studied (*n* = 32 studies), followed by men with prostate cancer (*n* = 8 studies) (Table 2).

**Table 2:** Overview of study characteristics of randomized controlled trials included in the meta-analysis (*n* = 71)

Study characteristic	Number of studies (*N*)
Cancer type	
Breast	32 [27, 29, 33, 41, 45, 47–50, 56, 60–62, 64, 66, 69, 71, 72, 78, 79, 81–83, 87–89, 93, 95–97, 99, 100]
Lung	4 [35, 40, 59, 70]
Non-Hodgkin’s lymphoma	1 [53]
Gynaecological	2 [36, 55]
Prostate	8 [44, 54, 57, 58, 65, 73, 77, 86]
Colon	1 [38]
Head and neck cancer	3 [46, 68, 74]
3+ cancer types	10 [28, 30, 34, 37, 42, 80, 90, 91, 94, 98]
Testicular	1 [67]
Lung + colorectal	1 [39]
Pancreas + peri-ampullary adenocarcinoma	1 [32]
Breast + colorectal	2 [52, 63]
Thyroid	1 [84]
Myeloproliferative neoplasm	1 [92]
Breast + gynaecological	1 [85]
Multiple myeloma	1 [43]
Gastrointestinal cancer	1 [51]
Sample size	
*N* ≤ 20	6 [28, 42, 49, 50, 70, 86]
*N* = 21–59	24 [30, 34–36, 38, 40, 41, 43, 44, 46, 55, 56, 58, 63, 64, 77, 81, 84, 85, 88, 91–93, 97]
*N* ≥ 60	41 [27, 29, 32, 33, 37, 39, 45, 47, 48, 51–54, 57, 59–62, 65–69, 71–74, 78–83, 87, 89, 90, 94–96, 98–100]
Stage of treatment during exercise intervention*	
During chemotherapy or radiotherapy	36 [28–30, 33, 35–37, 39, 41–43, 45–48, 50–52, 55, 60–62, 65, 67, 68, 72, 73, 77, 79, 80, 83, 91, 94, 98–100]
Post chemotherapy or radiotherapy	19 [27, 34, 38, 56, 57, 63, 64, 66, 69, 70, 74, 81, 85, 86, 89, 93, 95–97]
Active and post chemotherapy or radiotherapy	5 [32, 40, 54, 58, 82]
Timing of surgery with respect to the exercise intervention*	
Post-surgery	33 [27, 29, 32, 37, 38, 45, 47–49, 56, 59, 61, 63, 64, 66–71, 74, 77, 79, 81, 82, 84, 86, 88, 89, 93, 96, 97, 99]
Pre-surgery	3 [33, 36, 41]
Pre- and post-surgery	5 [30, 54, 60, 72, 78]
Intervention duration	
2 weeks	1 [36]
3 weeks	2 [53, 72]
4 weeks	3 [35, 51, 96]
6 weeks	5 [33, 43, 82, 95, 100]
8 weeks	13 [34, 38, 39, 46, 48, 55, 69, 70, 73, 78, 85, 89, 93]
10 weeks	3 [30, 52, 56]
12 weeks	22 [28, 31, 32, 40, 42, 44, 47, 49, 54, 58–60, 63, 64, 67, 74, 84, 86, 88, 92, 94, 99]
14 weeks	1 [80]
15 weeks	1 [79]
4 months	3 [62, 66, 77]
6 months	5 [61, 65, 81, 91, 98]
6.5 months	1 [45]
8 months	1 [71]
9 months	1 [97]
12 months	2 [27, 57]
50 weeks	1 [29]
Other†	6 [37, 41, 50, 68, 83, 90]
Exercise mode*	
Aerobic-based	15 [32, 33, 37, 40, 42–44, 50, 52, 62–64, 67, 91, 95]
Resistance-based	13 [29, 30, 49, 61, 74, 77, 80, 86, 87, 90, 94, 98, 99]
Mixed-mode	23 [27, 28, 35, 38, 39, 41, 45, 46, 54, 57, 58, 62, 65, 66, 69–71, 73, 83, 88, 89, 96, 97]
Yoga	12 [34, 36, 48, 56, 60, 78, 79, 82, 85, 92, 93, 100]
Other	9 [47, 51, 53, 55, 59, 68, 72, 81, 84]
Supervision	
Supervised	31 [28, 35, 37, 38, 41, 44–49, 54–56, 58, 59, 62, 66, 67, 69, 70, 72, 77, 81–84, 86, 87, 96, 97, 99]
Unsupervised	40 [27, 29, 30, 32–34, 36, 39, 40, 42, 43, 50–53, 57, 60, 61, 63–65, 68, 71, 73, 74, 78–80, 85, 88–95, 98, 100]
Definition of pain as defined in paper*	
Pain (not further specified)	45 [27–29, 33, 36–48, 50–53, 60–62, 65, 72, 73, 77–83, 87, 89–92, 94, 96–99]
Bodily pain	13 [32, 35, 54, 55, 57, 58, 63, 67, 70, 87, 88, 97, 100]
Joint/bone pain	3 [64, 86, 95]
Shoulder pain	5 [49, 56, 66, 69, 84]
Neck pain	1 [69]
Lymphoedema related	1 [93]
Neuropathic pain	8 [29, 30, 34, 71, 74, 85, 94, 98]
Oral pain	2 [46, 68]
Chronic pain	1 [38]
Postoperative pain	1 [59]
Pain when coughing/breathing	1 [59]
Affective pain	1 [36]
Pain measurement tools*	
EORTC QLQ C30- Pain symptom scale (2 items)	25 [28, 40, 41, 43–47, 51–53, 58, 60, 62, 65, 73, 74, 77, 79, 81–83, 90, 97, 99]
Numeric (Pain) Rating Scale (NPRS)	9 [29, 34, 36, 37, 39, 49, 61, 85, 91]
SF-36- Bodily pain subscale (2 items)	15 [32, 35, 42, 50, 54, 55, 57, 58, 63, 67, 70, 87, 88, 93, 97]
Visual Analogue Scale (VAS)	11 [32, 56, 59, 68, 69, 80, 86, 93, 95, 96, 98]
Neuropathic Pain Scale (NPS)	2 [30, 71]
Western Ontario and McMaster Uni OA Index (WOMAC)- Joint pain (5 items)	2 [64, 95]
The Penn Shoulder Scale- Pain subscale (3 items)	1 [66]
Chemotherapy-induced peripheral neuropathy assessment tool (CIPNAT)	1 [30]
Shoulder pain and disability index (SPADI)	1 [84]
Brief Pain Inventory (BPI)	7 [27, 38, 48, 72, 78, 87, 89]
NIH PROMIS Pain Intensity Short Form 3a	1 [92]
Total Neuropathy Score reduced (TNSr)	1 [94]
Functional Assessment of Cancer Therapy – Bone Pain (FACT-BP)	1 [86]
Prince Henry Hospital Pain Score (PHHPS)	1 [59]
Neuropathic pain in postsurgical patients (NeuPPS)	1 [29]
European Organization for Research and Treatment of Cancer (EORTC QLQ-CIPN15)	1 [94]
RAND-36 Measure of Health-Related Quality of Life	1 [33]
EORTC QLQ Head and Neck Module (H&N35)	1 [46]

*Five studies reported in eight papers included 2 exercise arms [31, 62, 71, 75, 76, 83, 87, 94], some reported multiple pain outcomes and pain measurement tools, and some did not report details on timing of surgery or stage of treatment with respect to the exercise intervention; therefore numbers do not always add up to *n* = 71 studies per row

^†^Duration of intervention: duration of chemotherapy [68, 83], individual participant’s length of hospital stay [37] during hospitalisation period of hematopoietic stem cell transplantation + 6 weeks after hospital discharge [90], not reported [68], “after enrolment, but before surgery” [41]

Most studies evaluated an exercise intervention during an active treatment period, with treatment comprising chemotherapy, radiotherapy or a combination of both. Less than 32% of studies evaluated exercise following treatment (i.e. all participants had completed all of their treatment for cancer) (Table 2). The predominant treatment type undertaken by participants across all cancer types was chemotherapy.

Fifteen studies included exercise interventions that were aerobic only, 13 were resistance only, 23 involved mixed-mode exercise (i.e. aerobic and resistance exercise) and 21 involved other exercise: 12 of those were yoga interventions (Table 3). Table S2 provides a more detailed description of exercise parameters. Intervention durations ranged between 2 weeks [36] and 12 months [57], with most interventions being 12 weeks or longer (*n* = 38/71, Table 2).

**Table 3:** Summary of exercise parameters evaluated in randomised controlled trials that were included in the meta-analysis

	Aerobic	Resistance	Other
Mode	Walking, cycling (stationary cycle or arm cycling), high intensity interval training (HIIT), treadmill; individual or group-based	Upper- and lower-body (including neck and head); machine, free-weight, range of motion and Theraband resistance exercises; individual or group-based	Yoga, Pilates, Dancing, Tai chi, Kyusho Jitsu, Qigong, Bouldering; individual or group-based
Intensity	Low–high	Low–high	Low–high
Session duration	20–60 min per session performed as continuous aerobic exercise; no session duration but a target of 10,000 steps a day; weekly total of 150 or more minutes	30–60 min per session	25–120 min
Frequency	2–7 sessions/week	2–7 sessions/week	1–7 sessions/week
Intervention duration	6 weeks–12 months	12 weeks–12 months	3–24 weeks
Progression	When specified: Aerobic: Increase in 1–2 metabolic equivalents per week; or, progressive increase in steps per day (up to 10,000) exercise time; and/or, increase intensity to maintain prescribed total exercise dose (mostly based on RPE or % of max heart rate). Resistance: Individualized exercise progression involving adding 1–2 new exercises per week; increase resistance weight by 5%–10%; increase repetitions. Generally, exercise was progressed if a given level of resistance could be performed with proper form, if individuals were able to perform more repetitions than the prescribed amount during a set, and/or based on results of retesting the one repetition maximum (1-RM)		
Indications to stop or reduce exercise	Participants received home exercise booklets that included when to stop or reduce exercise; received information of pain controlling strategies and when to seek out health professional; coaching emails with email feedback; reduced rate of progression and more supportive care when patients felt discomfort		

### Pain Outcomes

Across the 71 included studies, 13 (18%) studies reported pain as their primary outcome, 36 (51%) as a secondary or tertiary outcome, and a further 22 (31%) studies did not list pain as an outcome in their methods section. Only seven studies (10%) incorporated pain as an inclusion criterion, specified as patients with chemotherapy-induced peripheral neuropathy including symptoms of pain [30, 34, 85], neck and shoulder pain [69], shoulder dysfunction [84] and arthralgia [27, 95]. A further four studies (6%) purposely excluded patients with moderate-to-severe bone pain [86], concomitant conditions, such as previous low-back pain or musculoskeletal conditions [38], fibromyalgia or chronic pain disorders [61], or an average pain numeric rating scale score of > 6/10 [39]. The remaining 60 studies (85%) did not report any selection criteria related to pain.

Thirteen studies reported on bodily pain, eight on neuropathic pain, nine on musculoskeletal pain, two on oral pain, one on pain when coughing or breathing, one on chronic pain, one on lymphoedema-related pain, one on post-operative pain and one on affective pain. The remaining studies did not include any further specifications or definitions apart from ‘pain’ (Table 2).

All pain measurement tools were self-reported, measured through pain questionnaires or pain items/subscales in health-related questionnaires (i.e. validated quality of life questionnaires) (Table 2). The most commonly used pain assessment tools were the EORTC QLQ C30—Pain Symptom Scale (sum of two items on a four-point Likert Scale: ‘have you had pain?’, ‘did pain interfere with your daily activities?’) (*n* = 25 studies), the SF-36—Bodily Pain Subscale (sum of two items on a six-point Likert Scale: ‘how much bodily pain have you had during the past 4 weeks?’, ‘during the past 4 weeks, how much did pain interfere with your normal work?’) (*n* = 15 studies), the Visual Analogue Scale (*n* = 11 studies), the Numeric (Pain) Rating Scale (*n* = 9 studies) and the Brief Pain Inventory (BPI) (*n* = 7 studies) (Table 2). Across studies, the same types of pain were measured with different assessment tools. For example, studies measuring ‘shoulder pain’ used assessment tools including the Penn Shoulder Scale—Pain Subscale [66], the Shoulder Pain and Disability Index [84] and a Visual Analogue Scale [69]. Further, neuropathic pain was measured by the Neuropathic Pain Scale, the EORTC QLQ C30, chemotherapy-induced peripheral neuropathy (CIPN) symptom Likert Scale, the Functional Assessment of Cancer Therapy—General (FACT–G) and Numeric (Pain) Rating Scale (Table 2).

### Meta-analyses of Pain

**Fig. 2:**
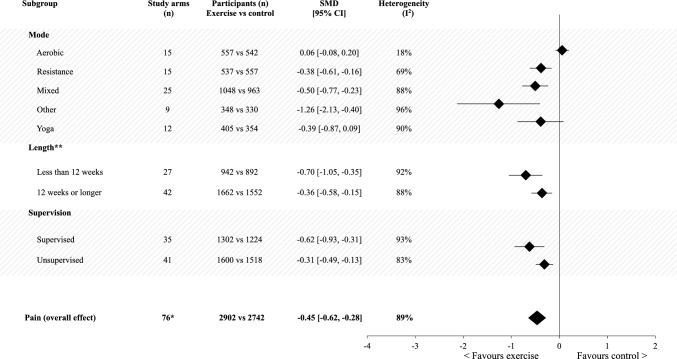
Meta-analysis of post-intervention pain outcomes between the exercise arm versus non-exercise/usual care arm, separated by exercise mode, length of intervention and supervision. *n = 76 intervention arms evaluated amongst 71 studies. **n = 7 intervention arms did not report the exact intervention duration. *CI* confidence interval,* n* number, *SMD* standardised mean difference

The meta-analysis of exercise compared with non-exercise/usual care groups post-intervention supported a reduction in pain favouring the exercise group, with a small effect size (SMD − 0.45; 95% CI − 0.62, − 0.28) (Table S3, Fig. 2).

The direction of effect was consistent and favoured exercise for all except one of the subgroup analyses related to intervention characteristics (i.e. mode, length of intervention, degree of supervision), with effect sizes ranging from small to large (effect size range − 0.31 to − 1.26; Fig. 2). The exception was for aerobic-only exercise interventions, with findings suggesting that aerobic-only exercise had no effect on pain compared to usual care (Fig. S1). Due to the lack of exercise intensity data, subgroup analyses on this intervention characteristics could not be performed.

**Fig. 3:**
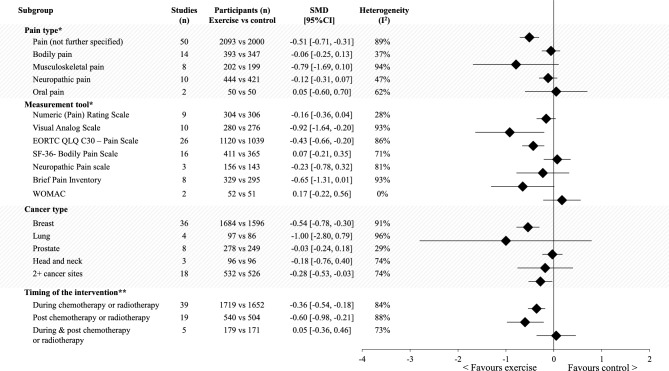
Meta-analysis of post-intervention pain outcomes between the exercise arm versus non-exercise/usual care arm, separated by pain category, measurement tools, cancer type and the timing of the exercise intervention with respect to cancer treatment. *Multiple data from one study (remembering five studies include two intervention arms [62, 71, 83, 87, 94]) can contribute to one or more of the categories within the above subgroups. **Treatment stage details during the exercise intervention could not be extracted for all studies. *CI* confidence interval, *n* number, *SMD* standardised mean difference

While trends support pain reduction through exercise for all cancer types studied, results were only supported statistically for studies involving women with breast cancer and for those studies including people diagnosed with one of more than two cancer types (Table S3). Further, subgroup analysis on the timing of the intervention with respect to treatment stage supported reductions in pain favouring the exercise group during and after chemotherapy or radiotherapy (effect sizes − 0.36 and − 0.60, respectively), but not for cohorts that included participants who were mixed during or after chemotherapy or radiotherapy (Fig. 3).

A moderate and significant effect favouring exercise was found for pain (not further specified) (SMD − 0.51; 95% CI − 0.71, − 0.31), but not for bodily pain, musculoskeletal pain or neuropathic pain (*p* ≥ 0.08). Further, small to large and significant effects favouring the exercise intervention were found for pain measured with the EORTC QLQ C30—Pain Scale, Visual Analogue Scale and the Brief Pain Inventory (effect sizes − 0.43, − 0.92 and − 0.65, respectively), but not for SF-36—Bodily Pain Scale, Numeric (Pain) Rating Scale, Neuropathic Pain Scale or the Western Ontario and McMaster Uni OA Index (*p* > 0.05) (Fig. 3, Table S3).

Sensitivity analyses showed that findings were consistent, irrespective of risk of bias score and selection criteria, but not for sample size (Table S3). Moderate effects were observed for RCTs with *n* ≥ 60 participants (SMD − 0.55; 95% CI − 0.78, − 0.33) and small effects for studies with *n* = 20–59 (SMD − 0.26; 95% CI − 0.50, − 0.02) participants, but not for studies with *n* < 20 (SMD 0.06; 95% CI − 0.31, 0.43) participants. Studies that included pain as an inclusion criterion showed a larger effect size (SMD − 0.79; 95% CI − 1.30, − 0.27) compared with those that listed pain as an exclusion criteria (SMD − 0.39; 95% CI − 0.37, − 0.04) and those that did not list pain within their eligibility criteria (SMD − 0.45; 95% CI − 0.64, − 0.25). However, all effect sizes were small to moderate and significant (*p* ≤ 0.03), favouring exercise.

### Strength of Evidence

Risk of bias assessed with the RoB 2 was mostly rated as having an overall score rated as high risk of bias (*n* = 38 papers), followed by some concerns (*n* = 25 papers) and low risk of bias (*n* = 11 papers) (Table S4). The domains mostly classified as being of concern were the ‘Selection of the reported result’ (*n* = 42, 57%) and ‘Measurement of the outcome’ (*n* = 39, 53%). This is likely because most studies measured pain as an item or subscale of a quality-of-life-related questionnaire, rather than by using a validated pain measurement tool.

The overall grade of the evidence of impact of exercise on pain outcomes after an exercise intervention was very low (Table S5).

## Discussion

Overall, findings for cancer-related pain management favoured participation in exercise compared with non-exercise or usual care interventions, with the overall effect size for pain being significant, albeit small. Further, subgroup analyses showed benefits for cancer-related pain, with small-to-large effect sizes, across multiple exercise modes and irrespective of intervention duration (i.e. < 12 weeks or 12 weeks and longer), degree of supervision provided throughout the intervention and timing with respect to cancer treatment (that is, benefits were observed during and post-cancer treatment). While trends favouring exercise were observed for all cancer types, pain types and most pain measurement tools, results were only supported statistically for women with breast cancer, pain (not further specified) and pain when assessed with the EORTC QLC C30—Pain Scale, the Visual Analogue Scale and the Brief Pain Inventory.

Two previous systematic reviews have evaluated exercise interventions aimed at reducing shoulder pain in women with breast cancer [20, 21]. Although neither of these reviews involved the conduct of a meta-analysis, the authors concluded that exercise therapy [21], including targeted physiotherapy-based interventions [20] might be effective for reducing and managing shoulder pain. Specifically, Giacalone et al. [20] stated that musculoskeletal pain may be managed through active exercises, joint and tissue mobilisation, and that neuropathic pain might be managed through aerobic and strengthening exercises, both while supervised by experienced physiotherapists. Our review included exercise types beyond physiotherapy-based resistance and mobility exercises that target specific muscle and/or joint groups. As such, the results of this review support and extend on the previous review evidence base [20, 21] through findings that support benefit through unsupervised as well as supervised exercise, and exercise modes, including general resistance only, mixed resistance and aerobic, yoga and other types including martial arts, dance, pilates and bouldering. Of note, our null findings were in relation to the effect of aerobic-only exercise interventions compared with usual care groups, potentially suggesting that exercise modes that specifically target musculoskeletal strength and/or endurance (such as resistance, mixed mode, yoga or other modes) are particularly beneficial (and potentially necessary) for pain management in cancer care. Uncertain effects of aerobic exercise on pain outcomes have also been reported in some non-cancer chronic pain populations, such as fibromyalgia [101]. In contrast, in other populations, such as chronic low-back pain, aerobic exercise has been shown to improve pain [102]. More research is clearly needed to better understand the relationship between exercise mode and pain response.

While findings from this meta-analysis support pain reductions through exercise compared with non-exercise/usual care interventions, the magnitude and certainty of the effect according to pain type (nociceptive, neuropathic, visceral, musculoskeletal), location/site of pain (shoulder, hands, feet, breast), duration of pain (acute, subacute, chronic) and severity of pain (level of intensity) remain unclear. None of the included RCTs specified all of these important aspects of pain. To further advance understanding of cancer-related pain and treatment strategies, future research would benefit from a more comprehensive description of pain type, duration, site, severity/intensity of pain, and presence and type of concurrent pain treatment and/or medication usage (as these influence pain and pain fluctuations). Further, cancer-related pain outcomes were measured using a wide range of self-reported tools including pain-specific questionnaires and pain items or subscales from quality-of-life-related questionnaires. Some tools measured pain intensity on a Visual Analogue Scale or Numeric Rating Scale, but the vast majority of studies used tools that included a pain subscale score combining questions related to pain intensity (for example, SF-36: ‘how much bodily pain have you had?’) and how much pain interfered with daily living, activities or work (for example, SF-36: ‘how much did pain interfere with your normal work?’). This heterogeneity in pain assessments, in addition to the poor description of cancer-related pain, likely contributed to the wide confidence intervals observed within specific subgroup analyses. Nonetheless, our findings are derived from data collected via 71 RCTs including 5877 participants, and revealed pain reductions through participation in exercise.

The mechanisms through which exercise benefits pain are likely multi-factorial (involving at least biological, physical and psychosocial factors) and complex, with differences between people with and without pain, among those with acute, subacute and chronic pain, and for those with different types of pain (for example, nociceptive, nociplastic and neuropathic pain). There is extensive evidence in non-cancer chronic pain conditions and chronic diseases demonstrating the benefits to the cardiovascular, respiratory and musculoskeletal system through exercise, as well as to mood and overall quality of life, and evidence suggests these benefits contribute to reductions in pain [103, 104]. There is also evidence, albeit less consistent, that suggests exercise can reduce pain through reductions in nervous system sensitivity at spinal and supraspinal levels in non-cancer chronic pain conditions, such as chronic low back pain and fibromyalgia. However, where and in which pathways these changes occur for different types of pain remains unclear and warrants future investigation [105, 106].

How people think about their pain and the assumptions they hold also influence their pain perceptions and their behavioural response to pain [107]. For example, some people avoid exercise or physical activity as they perceive that exercise increases pain or is harmful for the body. While the findings of this systematic review with meta-analysis suggest otherwise, it is nonetheless important to acknowledge that the results are drawn from studies that have measured pain as an outcome of interest. Exercise-related adverse effects, of which pain may be one of several﻿, are poorly measured and reported [108] in exercise oncology trials. Improvements in harms assessment and reporting in future exercise oncology research will further benefit our understanding of the potential for exercise to cause or exacerbate pain. In healthy people, pain catastrophising and fear of pain appear to decrease the hypoalgesic effects of exercise [109]. In people with cancer-related pain, a systematic review showed associations between higher levels of pain and increased psychological distress (*n* = 14 studies, strong level of evidence), and between higher levels of pain and decreased levels of social activities and social support (*n* = 8 studies, moderate level of evidence) [7]. Understanding the inter-relationships among psychological distress and social support, exercise and pain also merits future research attention and could aid in improving cancer-related pain management [110].

The strengths of this systematic review include the screening, extraction and risk of bias ratings being conducted by two independent authors with disagreements resolved by a senior author, and the inclusion of data collected from people with 21 different cancer types. Several limitations also need to be considered. Eleven out of 74 papers (from 71 studies) were graded as having a low risk of bias, but 38 papers (52%) were graded as having a high risk of bias. The inclusion of all types of cancer-related pain, irrespective of origin, for all cancer types could be viewed as both a strength and limitation of this meta-analysis. Only seven studies (10%) included pain as an inclusion criterion, while four studies (6%) specifically excluded people with pain, which potentially reduces the capacity to observe an effect on pain through exercise. Nonetheless, this also suggests that our findings are likely in the conservative direction. Further, the results of sensitivity analyses, which considered the effect of including studies with high risk of bias, and studies that included participants with pain, excluded participants with pain or did not report pain as eligibility criteria, remained similar. While medication use for pain was unknown for participants in the included trials, presumably randomisation ensured balance between the intervention and control groups for the outcome variable.

## Conclusions

This is the first comprehensive systematic review with meta-analysis that evaluated the effect of exercise on cancer-related pain, and considered all exercise modes, length of intervention, degree of supervision, cancer types, pain types, measurement tools and timing of the intervention (during and post-cancer treatment). The findings provide support that exercise participation does not worsen cancer-related pain and that it could be beneficial for the wider cancer population, including during and following active treatment for cancer in the management of cancer-related pain. Our findings provide confidence to health professionals in their prescription of exercise to people with cancer-related pain, and their promotion of participating in a range of exercise modes, including under unsupervised conditions. However, further advancements in understanding require inclusion of more diverse cancer populations and improvements in the reporting of types of pain (i.e. visceral, neuropathic, nociceptive), as well as on the location, duration and intensity of pain.

## Supplementary Information

Below is the link to the electronic supplementary material.

Supplementary file1 (PDF 693 KB)

## Declarations

### Availability of data and material

Data are available on request from the authors.
