# Supplementary material for: The Effect of Exercise on Pain in People with Cancer: A Systematic Review with Meta-analysis
Source: Sports Med. 2023 May 22;53(9):1737–52. doi: 10.1007/s40279-023-01862-9 (PMC10432370; doi:10.1007/s40279-023-01862-9)
Supplement: Supplementary file 1 — Supplementary file1 (PDF 693 KB) [file 40279_2023_1862_MOESM1_ESM.pdf]

Supplementary Material

Article title: The effect of exercise on pain in people with cancer: a systematic review with meta-analysis

Journal name: Sports Medicine

Author names: Melanie Louise Plinsinga[1], Ben Singh[2], Grace Laura Rose[3], Briana Clifford[3,4], Tom George Bailey[3], Rosalind Renee Spence[1], Jemma Turner[1], Michel Willem Coppieters[1,5], Alexandra Leigh McCarthy[3], Sandra Christine Hayes[1]

Affiliations:

1. School of Health Sciences and Social Work, Menzies Health Institute Queensland, Griffith University, Brisbane and Gold Coast, Australia.
2. Allied Health and Human Performance, Alliance for Research in Exercise, Nutrition and Activity, University of South Australia, Adelaide, Australia.
3. School of Nursing, Midwifery and Social Work, The University of Queensland, and Mater Research Institute, Brisbane, Australia.
4. School of Health Sciences, University of New South Wales, Sydney, Australia.
5. Faculty of Behavioural and Movement Sciences, Amsterdam Movement Sciences – Musculoskeletal Health Program, Vrije Universiteit Amsterdam, Amsterdam, The Netherlands

E-mail address of corresponding author: m.plinsinga@griffith.edu.au

Study or Subgroup	Experimental Mean	Experimental SD	Experimental Total	Control Mean	Control SD	Control Total	Weight	Std. Mean Difference IV, Random, 95% CI
**1.2.1 Aerobic**								
Adams 2018	52.5	7.3	35	52.8	7.9	28	6.5%	−0.04 [−0.54, 0.46]
Backman 2014	15.2	22.2	39	18.3	27	38	7.7%	−0.12 [−0.57, 0.32]
Bade 2021	13.884	4.79	20	13.47	5.29	20	4.4%	0.08 [−0.54, 0.70]
Forner 205	65.56	35.81	9	70.63	21.66	8	2.0%	−0.16 [−1.11, 0.79]
Hacker 2022	18.6	19.4	17	21.1	20.4	15	3.6%	−0.12 [−0.82, 0.57]
Hammer 2021	2.6	3.7921	15	1.5	1.8098	18	3.6%	0.37 [−0.32, 1.06]
Heimann 2022	70.49	24.8	139	69.5	23.6	148	18.5%	0.04 [−0.19, 0.27]
Kang 2022	15.3	14.4	25	10.7	15.1	25	5.3%	0.31 [−0.25, 0.86]
Mijwel 2017	17.35	19.08	70	27.95	30.3	60	11.2%	−0.42 [−0.77, −0.07]
Nyrop 2017	4.47	2.53	31	4.82	2.44	31	6.5%	−0.14 [−0.64, 0.36]
Rastogi 2020	51.1	11.6	26	50.1	11.3	24	5.4%	0.09 [−0.47, 0.64]
Rogers 2009	3.03	2.98	21	2.08	2.14	20	4.5%	0.36 [−0.26, 0.98]
Steffens 2022	4.69	2.94	47	4.1	3.02	49	9.1%	0.20 [−0.20, 0.60]
Sturgeon 2022	65.3	22.4	9	50.7	25.7	10	2.1%	0.58 [−0.35, 1.50]
Yeo 2012	50.33	9.25	54	46.38	7.995	48	9.4%	0.45 [0.06, 0.85]
**Subtotal (95% CI)**			**557**			**542**	**100.0%**	**0.06 [−0.08, 0.20]**
Heterogeneity: $Tau^2 = 0.01$; $Chi^2 = 17.14$, df = 14 (P = 0.25); $I^2 = 18\%$								
Test for overall effect: Z = 0.82 (P = 0.41)								
**Total (95% CI)**			**557**			**542**	**100.0%**	**0.06 [−0.08, 0.20]**
Heterogeneity: $Tau^2 = 0.01$; $Chi^2 = 17.14$, df = 14 (P = 0.25); $I^2 = 18\%$								
Test for overall effect: Z = 0.82 (P = 0.41)								
Test for subgroup differences: Not applicable								

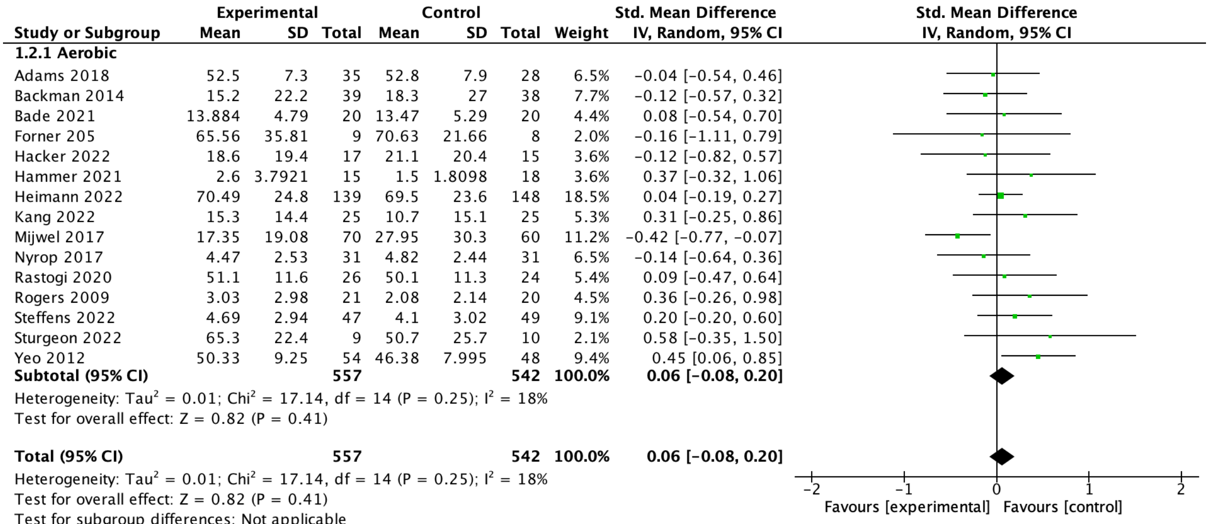

Figure S1. Meta-analysis of post-intervention pain outcomes between the exercise arm versus non-exercise/usual care arm, for aerobic exercise mode only. Abbreviations: CI: confidence interval; Std: standardised; df: degrees of freedom.

Table S1. Full search terms for each electronic database, conducted up to 10 January 2023.

Table S1A. Search terms PubMed.

	**"pain"**	**"physical activity"/"exercise"**	**"cancer"**
	"Pain"[MeSH Terms] OR "Pain"[Text Word] OR "Cancer Pain"[MeSH Terms] OR "Cancer Pain"[Text Word] OR "Pain Measurement"[MeSH Terms] OR "Pain Measurement"[Text Word]	"Exercise"[MeSH Terms] OR "Exercise"[Title/Abstract] OR "physical activit*"[Title/Abstract] OR "aerobic exercise"[Title/Abstract] OR "resistance training"[Title/Abstract] OR "active lifestyle*"[Title/Abstract] OR "lifestyle intervention"[Title/Abstract] OR "Sports"[MeSH Terms] OR "swim*"[Title/Abstract] OR "yoga"[Title/Abstract] OR "pilates"[Title/Abstract] OR "weightlifting"[Title/Abstract] OR "Exercise Therapy"[MeSH Terms] OR "Exercise Therapy"[Title/Abstract] OR "Exercise rehabilitation"[Title/Abstract] OR "sport*"[Title/Abstract] OR "sedentary behavior"[MeSH Terms] OR "sedentary lifestyle"[Title/Abstract] OR "physical inactivity"[Title/Abstract]	"Neoplasms"[MeSH Terms] OR "neoplasm*"[Title/Abstract] OR "Cancer"[Title/Abstract] OR "malignan*"[Title/Abstract]
**FINAL SEARCH**	("Pain"[MeSH Terms] OR "Pain"[Text Word] OR "Cancer Pain"[MeSH Terms] OR "Cancer Pain"[Text Word] OR "Pain Measurement"[MeSH Terms] OR "Pain Measurement"[Text Word]) AND ("Exercise"[MeSH Terms] OR "Exercise"[Title/Abstract] OR "physical activit*"[Title/Abstract] OR "aerobic exercise"[Title/Abstract] OR "resistance training"[Title/Abstract] OR "active lifestyle*"[Title/Abstract] OR "lifestyle intervention"[Title/Abstract] OR "Sports"[MeSH Terms] OR "swim*"[Title/Abstract] OR "yoga"[Title/Abstract] OR "pilates"[Title/Abstract] OR "weightlifting"[Title/Abstract] OR "Exercise Therapy"[MeSH Terms] OR "Exercise Therapy"[Title/Abstract] OR "Exercise rehabilitation"[Title/Abstract] OR "sport*"[Title/Abstract] OR "sedentary behavior"[MeSH Terms] OR "sedentary lifestyle"[Title/Abstract] OR "physical inactivity"[Title/Abstract]) AND ("Neoplasms"[MeSH Terms] OR "neoplasm*"[Title/Abstract] OR "Cancer"[Title/Abstract] OR "malignan*"[Title/Abstract])		
**Hits**	2,188		

Table S1B. Search terms Embase, limited to adults.

	**"pain"**	**"physical activity"/"exercise"**	**"cancer"**
	'pain'/exp OR 'cancer pain'/exp OR 'pain measurement'/exp OR 'pain' OR 'cancer pain' OR 'pain measurement'	'exercise'/exp OR 'physical activity'/exp OR 'sport'/exp OR 'kinesiotherapy'/exp OR 'sedentary lifestyle'/exp OR 'dance therapy'/exp OR 'recreational therapy'/exp OR exercise:ab,ti OR 'aerobic exercise':ab,ti OR 'anaerobic exercise':ab,ti OR 'aquatic exercise':ab,ti OR pilates:ab,ti OR 'resistance training':ab,ti OR 'active lifestyle':ab,ti OR 'lifestyle intervention':ab,ti OR 'physical activit*':ab,ti OR swimming:ab,ti OR 'weight lifting':ab,ti OR yoga:ab,ti OR kinesiotherapy:ab,ti OR 'exercise therapy':ab,ti OR 'exercise rehabilitation':ab,ti OR 'sedentary lifestyle':ab,ti OR 'sedentary behavior':ab,ti OR 'physical inactivity':ab,ti OR 'recreation therapy':ab,ti OR 'recreational therapy':ab,ti	'malignant neoplasm'/exp OR 'malignant neoplasm':ab,ti OR 'cancer':ab,ti
**FINAL SEARCH**	('pain'/exp OR 'cancer pain'/exp OR 'pain measurement'/exp OR 'pain' OR 'cancer pain' OR 'pain measurement') AND ('exercise'/exp OR 'physical activity'/exp OR 'sport'/exp OR 'kinesiotherapy'/exp OR 'sedentary lifestyle'/exp OR 'dance therapy'/exp OR 'recreational therapy'/exp OR exercise:ab,ti OR 'aerobic exercise':ab,ti OR 'anaerobic exercise':ab,ti OR 'aquatic exercise':ab,ti OR pilates:ab,ti OR 'resistance training':ab,ti OR 'active lifestyle':ab,ti OR 'lifestyle intervention':ab,ti OR 'physical activit*':ab,ti OR swimming:ab,ti OR 'weight lifting':ab,ti OR yoga:ab,ti OR kinesiotherapy:ab,ti OR 'exercise therapy':ab,ti OR 'exercise rehabilitation':ab,ti OR 'sedentary lifestyle':ab,ti OR 'sedentary behavior':ab,ti OR 'physical inactivity':ab,ti OR 'recreation therapy':ab,ti OR 'recreational therapy':ab,ti) AND ('malignant neoplasm'/exp OR 'malignant neoplasm':ab,ti OR 'cancer':ab,ti)		
**Hits**	4,764		

Table S1C. Search terms Scopus limited to articles, journals.

	**"pain"**	**"physical activity"/"exercise"**	**"cancer"**
	ALL (pain) OR ALL ("cancer pain") OR ALL ("pain measurement")	TITLE-ABS-KEY (exercise) OR TITLE-ABS-KEY ("resistance training") OR TITLE-ABS-KEY ("lifestyle intervention") OR TITLE-ABS-KEY (yoga) OR TITLE-ABS-KEY (pilates) OR TITLE-ABS-KEY (weightlifting) OR TITLE-ABS-KEY ("exercise therapy") OR TITLE-ABS-KEY ("exercise rehabilitation") OR TITLE-ABS-KEY ("recreation therapy") OR TITLE-ABS-KEY ("aquatic exercise")	TITLE-ABS-KEY ("malignant neoplasm*") OR TITLE-ABS-KEY (cancer)
**FINAL SEARCH**	(ALL (pain) OR ALL ("cancer pain") OR ALL ("pain measurement")) AND (TITLE-ABS-KEY (exercise) OR TITLE-ABS-KEY ("resistance training") OR TITLE-ABS-KEY ("lifestyle intervention") OR TITLE-ABS-KEY (yoga) OR TITLE-ABS-KEY (pilates) OR TITLE-ABS-KEY (weightlifting) OR TITLE-ABS-KEY ("exercise therapy") OR TITLE-ABS-KEY ("exercise rehabilitation") OR TITLE-ABS-KEY ("recreation therapy") OR TITLE-ABS-KEY ("aquatic exercise")) AND (TITLE-ABS-KEY ("malignant neoplasm*") OR TITLE-ABS-KEY (cancer))		
**Hits**	5,394		

Table S1D. Search term CINAHL, SPORTDiscuss.

	**"pain"**	**"physical activity"/"exercise"**	**"cancer"**
	S17. (MH "Pain+") OR (MH "Pain Measurement") OR (MH "Cancer Pain") S18. TX "pain" OR TX "cancer pain" OR TX "pain measurement" S19. S17 OR S18	S1. (MH "Exercise+") OR (MH "Resistance Training") OR (MH "Therapeutic Exercise+") OR (MH "Exercise Therapy: Muscle Control (Iowa NIC)") OR (MH "Exercise Therapy: Joint Mobility (Iowa NIC)") OR (MH "Exercise Therapy: Balance (Iowa NIC)") OR (MH "Exercise Therapy: Ambulation (Iowa NIC)") OR (MH "Aerobic Exercises") OR (MH "Aquatic Exercises") OR (MH "Anaerobic Exercises" S2. AB exercise OR AB "physical activit*" OR AB "active lifestyle" OR AB "lifestyle intervention" OR AB "resistance training" OR AB "therapeutic exercise" OR AB "exercise therapy" OR AB "exercise rehabilitation" OR AB "aerobic exercise" OR AB "aquatic exercise" OR AB "anaerobic exercise" S3. MH "Sports+") OR (MH "Running") S4. (MH "Yoga+") S5. (MH "Pilates") S6. (MH "Walking+") S7. (MH "Dance Therapy") S8. (MH "Recreation Therapy (Iowa NIC)") OR (MH "Recreational Therapy") S9. (MH "Life Style, Sedentary+") S10. AB yoga OR AB pilates OR AB run* OR AB jog* OR AB walk* OR AB sport* OR AB move* OR AB swim* OR AB weightlifting OR AB bicycl* S11. AB "dance therap*" OR AB danc* OR AB "recreation therapy" OR AB "sedentary behavior" OR AB "sedentary lifestyle" OR AB "physical inactivity" S14. S1 OR S2 OR S3 OR S4 OR S5 OR S6 OR S7 OR S8 OR S9 OR S10 OR S11	S20. AB malignant neoplasm OR AB cancer
**FINAL SEARCH**	S19 AND S14 AND S20		
**Hits**	1,527		

Table S1E. Search term Cochrane Library. No limits.

	"pain"	"physical activity"/"exercise"	"cancer"
	#1 MeSH descriptor: [Pain] explode all trees<br>#2 MeSH descriptor: [Pain Measurement] explode all trees<br>#3 MeSH descriptor: [Cancer Pain] explode all trees<br>#4 ("pain" OR "pain measurement" OR "cancer pain")<br>#5 #1 OR #2 OR #3 OR #4	#6 MeSH descriptor: [Exercise] explode all trees<br>#7 MeSH descriptor: [Sports] explode all trees<br>#8 MeSH descriptor: [Exercise Therapy] explode all trees<br>#9 MeSH descriptor: [Recreation Therapy] explode all trees<br>#10 ("exercise" OR "physical activit*" OR "resistance training" OR "active lifestyle" OR "lifestyle intervention" OR "swimming" OR "yoga" OR "pilates" OR "weightlifting" OR "exercise therapy" OR "exercise rehabilitation" OR "sedentary behavior" OR "sedentary lifestyle" OR "physical inactivity" OR "recreation therapy"):ti,ab,kw<br>#11 #6 OR #7 OR #8 OR #9 OR #10	#12 MeSH descriptor: [Neoplasms] explode all trees<br>#13 ("malig nant neoplasm*" OR "cancer"):ti,ab,k w<br>#14 #12 OR #13
**FINAL SEARCH**	#16 #5 AND #11 AND #14		
**Hits**	944		

Table S2. Description of exercise parameters (mode, intensity, duration) evaluated in the randomised controlled trials included in the meta-analysis (n=71 studies).

**Intervention details from the randomised, controlled trials**				
**Aerobic (n=15)**	**Resistance (n=13)**	**Mixed-mode (aerobic + resistance) (n=23)**	**Other (n=9)**	**Yoga (n=12)**
Walking; F: daily x 12 wks; I: ns; D: until step count is met (target 10,000 steps a day) [40]	Therapeutic home exercises to restore full upper limb range of motion; F: 3x sess/day x 6 months; I: ns; D: ns [61]	Walking and upper (5 exercises) and lower body (5 exercises) strength program; F: 2-4 sess/wk x 8 wks; I: 10 reps per exercise, usage of resistance bands (ns), moderate intensity (ns). D: ns [39]	Qigong (ns); F: daily x 3 wks; I: ns; D: 25 min [53]	Dru Yoga (breathing awareness 15 min, energy block 15 min, asana 30 min, relaxation 15 min); F: daily x 12wks; I: ns; D: 75 at hospital sess (1sess/week), minimum 5min at home/sess [60]
Walking; F: daily x 12 wks; I: moderate, progression based on fitness at baseline; D: 20-40min/day [32]	3 lying down exercises (ankle motion, hip abduction, leg raise) and 5 sitting exercises (abduction/adduction, wrist motion, elbow flexion/extension, knee flexion/extension, toe tapping); F: daily x 10 wks; I: ns; D: 30min/sess [30]	Resistance (5 upper body and 3 lower body strength exercises) and aerobic (cycling, walking or jogging) exercise program; 4 sess/wk x 12 mnths; I: resistance 2-4 sets x 6-12 reps progressing from 12RM to 6RM, aerobic 70-85% HRmax and RPE of 11-13 on 6-20 Borg scale; D: resistance ns, aerobic 20-30 min [57]	Wall climbing aka bouldering; F: 2 sess/wk x 8 wks; I: ns progression based on individual response by varying route difficulty, time set to complete the route or height achieves; D: 120 min/sess [55]	Hatha yoga; F: 2sess/wk x 8wks; I: ns (beginner level); D:60min [56]
Walking; F: daily x 10wks; I: ns; D; ns, walking target was 10,000 steps/day (approximately 8km/day) [52]	Resistance and Pilates (ns); F:3sess/wk x 50wks; I: loading at 25RM, 2-3sets x 20reps; D: 30min [29]	RTHIIT intervention: 10 resistance exercises targeting major muscle groups using weight equipment, body mass, free weight dumbbells, or barbells (leg press, bicep curls, squat jumps, triceps extensions, lunges, bench press, sit-up or Russian-weighted abdominal twist, shoulder press, back extensions) and aerobic exercise on cycle ergometer; F: 2sess/wk x 16wks; I: resistance 2-3sets x 8-12reps at 70% 1RM progressing to 80% 1RM (new 1RM test if >12reps), aerobic 3x3min bouts on 16-18 Borg scale, 1 min active low intensity recovery in between; D: 60min/sess [31, 62, 75, 76]	Yang 24-form Tai Chi; F: daily x 12wks; I: ns; D: 60min (10min warm up, 40min practice, 10min cool down) [59]	Yoga exercises (diaphragmatic breathing, systematic relaxation, alternate nostril breathing, neck and shoulder exercises); F: 2sess/day x 15wks; I: low; D:ns [79]
Walking; F: 3-5sess/wk x 12wks; I: moderate; D: 150min/wk, duration/sess progressed over time (ns) [64]	7 flexibility exercises for head, neck, shoulder, 3 range of motion exercises for tongue, lips, jaw, 5 lymphedema exercises (if facial lymphedema is present); F: 3sess/wk x 12 wks; I: ns; D: ns [74]	8 resistance exercises (leg press, lunges, knee extension, knee flexion, chest press, seated row, triceps extension, bicep curl) and walking/running; F: 3sess/wk x 16wks; I: resistance 60-80% 1RM (progression through increase reps and progressive overload 10%), aerobic 65-80% Hrmax (new Hrmax every 4 weeks); D: 50-80min [66]	Physiotherapy supervised Pilates (mat and TheraBand exercises); F: 3sess/wk x 12wks; I: moderate to vigorous (ns); D: 40-45min [84]	Yoga exercises (yoga postures, breathing exercise including nostril breathing, followed by a moving meditation practice); F: 3sess/wk x 6wks; I:ns; D: 60min [82]
AT-HITT intervention: 20 min on cycle ergometers, elliptical ergometer, treadmill followed by HIIT aerobic exercise on cycle ergometer; F: 2sess/wk x 16wks; I: 20min moderate then aerobic 3x3min bouts on 16-18 Borg scale, 1 min active low	Nine machine and free-weight exercises (smith machine half squat, leg press, smith machine standing calf raises, knee flexion, knee extension, chest press, seated row, seated shoulder press, biceps curl); F: 3sess/wk x 16wks; I:60-80%1RM, 2-3sets x 10reps; D:ns [77]	8 upper and lower body resistance exercises and aerobic (cycling, jogging, walking, rowing); F: 2sess/wk x 12wks; I:	Dance (stretching, relaxation exercises, movement games, rhythmic body movement to exercise upper extremity, improvisational dance and movement); F: 2sess/wk x 3wks; I: moderate to vigorous (ns); D: 90min [72]	Yoga (gentle postures 40min, breathing techniques 10min, meditation 25min, application yogic principles to optimal coping 20min, group
	3 isometric exercises targeting vertebral column muscles; F: 3-		Kyushu Jitsu (aspects of martial arts, self-defence, body related	

intensity recovery in between; D: 60min/sess [31, 62, 75, 76]<br><br>Walking; F: daily x 12wks; I: ns, gradually increase steps/day and min/wk; D: 150min/wk and 10,000 steps/day [63]<br><br>HIIT (4 intervals separated by active recovery); F: 3sess/wk x 12wks; I:75%VO2max during four 4 minute high intensity intervals, 3 minute active recovery 5-10% below ventilatory threshold?; D:35min [67]<br><br>Walking; F:3sess/wk x 6mnths; I:80-100% max RPE; D:30min [91]<br><br>Walking; F: 3-4sess/wk x 6wks; I: moderate intensity (ns); D:150min/wk [95]<br><br>Aerobic activity (ns); F: daily x 5-6wks; I: medium intensity; D: 30mins [33]<br><br>Walking; F: 1sess/wk x 12wks; I: ns; D: ns [42]<br><br>HIIT on treadmill (5-8 intervals warm up, high intensity walking/jogging,	5sess/wk x 14wks; I:ns; D:30-45min [80]<br><br>6 upper body resistance exercises (chest press, seated row/lat pulldown, shoulder press/lateral raise, bicep curl, triceps extension, wrist curl); F: 2sess/wk x 12wks; I: high load group 1-4sets of 75-85% of 1RM using 6-10RM, low load group 1-4sets of 55-56% of 1RM using 15-20RM; D:60min [87]<br><br>Resistance training of the paravertebral muscles (ns); F:3-5sess/wk x 6mnths; I: ns; D: 30min [98]<br><br>SMT group balance and proprioception training, RT group machine based resistance training; F: 3sess/wk x 12wks; I: SMT group guided by RPE, RT group 70-80% 1RM at RPE 14-16. D:35-45min [94]<br><br>Machine based resistance exercises; F: 2sess/wk x 12wks; I: 3 sets x 8-12reps at 60-80% 1RM (progression via 1RM); D: 60min [99]<br><br>8 upper and lower body resistance exercises targeting the major muscle groups; F: 2sess/wk x 12wks; I: 2-4sets, 12-8RM; D:60min [86]<br><br>11 exercises using resistance bands and body weight (chest fly, biceps curl, triceps extension, shoulder shrug, shoulder upright row, shoulder lateral raise, knee flexion, and knee extension, wall push ups, squats, bed sit-ups); F:	resistance 60-85% 1RM (5-10% incremental increase), aerobic 70-85% Hrmax (high RPE); D: 60min [54]<br><br>7 lumbopelvic stabilization exercises, aerobic exercise (walk or run) and stretching exercises; F: 3sess/wk x 8wks; I: resistance 1-3 sets x 8-10reps, aerobic based on RPE (ns), stretching low, progression through increased intensity, volume and motor control demand; D: 90min max (aerobic 10-25min, stretching 10 min) [38]<br><br>8 resistance (chest press, seated row, shoulder press, triceps extension, leg press, leg extension, leg curl and abdominal crunches), aerobic (cycling, walking, jogging), flexibility exercises; F: 2sess/wk x 12ks; I: resistance 60-80% 1RM 2-4sets x 6-12reps, aerobic 65-80% Hrmax; D: resistance ns, aerobic 15-20min, flexibility 15-20min [58]<br><br>6 resistance and HIIT; F: 2-3sess/wk x 6mnths; I: resistance 6-12RM, aerobic 60-85% Hrmax; D: 20-40min [65]<br><br>OM group: walking and resistance, OT group: aerobic (walking, running, HIIT) and resistance; F: 5sess/wk x duration of chemotherapy; I: OM moderate based on RPE, OT moderate to vigorous based on Hrmax, resistance progression 1RM testing every 3 weeks; D: 30min [83]<br><br>Water based resistance and aerobic exercises; F: 3sess/wk x 8wks; I: low intensity in 32 degree pool, intensity gradually increased by physical therapist; D: 60min (10min warm up, 35min aerobic, endurance and core stability, 15min cool down) [69]<br><br>Upper and lower limb resistance (ns) and aerobic (treadmill) exercises; F: 5sess/wk x 12wks; I: resistance 1-3 sets (sets increased progressively) x 10-15reps until failure, aerobic 5min at 40%HRR, 40min at 75% HRR, 5min at 40%HRR; D: resistance ns, aerobic 50min [28]<br><br>Aerobic and resistance exercises (ns); F: 4sess/wk x 8mnts; I: moderate to vigorous (ns); D:45min [71]	cognition, breathing exercises, medication, stretching and physical strengthening); F: 2sess/wk x 24wks; I:ns; D:90min [81]<br><br>Mastecation training (10x mount opening and closing, 10x right lateral excursion, 10x left lateral excursion, 10x mandibular protrusion, 5min alternating bilateral chewing with hyperboloid devise; F: 4sess/daily x ns; I: new hyperboloid device each week; D: ns [68]<br><br>Baduanjin that included eight standardised postures (both hands to sky, both hands side like shooting vulture, raise single arm, look back, sway head and shake the tail, clench fists, touch toes by hands with flexion of hip and extension of knee joint, rise and bounce of toes seven times); F: 2sess/wk x 12 wks; I: ns; D: 90min (10min warm up, 70mins Baduanjin, 10min cooldown). [47]<br><br>Qigong exercise focussing on monkey frolic postures; F: 5sess/wk x 4 wks; I: ns; D: 15-18min [51]	discussions 25 min); F:1sess/wk x 8wks; I:ns; D:120min [78]<br><br>Home based online yoga; F:1sess/wk x 12wks; I: moderate to vigorous (ns); D: 20-60min (time gradually increased) [92]<br><br>Yoga (breathwork, modifiable postures (asanas) to improve circulation, aerobic capacity, joint strength and mobility, spinal flexibility and tone, core strength, balance and proprioception); F: daily x 8wks; I: ns D:60min [85]<br><br>Yoga (movements synchronized with breathing, maintenance in postures forward, backwards and side-bending asanas in sitting and standing positions, cobra, crocodile, and half-shoulder stand with support, deep relaxation techniques); F: 2-7sess/wk x 6wks; I:ns; D:60min [100]<br><br>Yoga (breathing practices, physical postures, meditation and relaxation techniques according to the Satyananda Yoga tradition); F: daily x 8wks; I:ns; D: 45-90min [93]<br><br>Yoga (guided breathing exercises, upper and lower

active recovery); F: 1sess/wk x 12 wks; I: warm up at 60% VO2peak, interval at 95-95%VO2peak, active recovery at 40% VO2peak; D: 55-82min (intervals 45-72min, cool down 10min) [44]<br><br>Walking; F: daily sess/wk x length of hospital stay; I: ns, participants progressed based on daily step count where step count was increased by 50% each day until patient discharged from hospital; D: ns [37]<br><br>Aerobic exercises as circuit training (marching in place, side touches, side taps, knee lifts, front/back kicks, hamstring curls, walking forward and backward, jogging in place, skater hops, grapevines, arm swings, overhead reaches, clapping, front presses and arm rows); F: 1sess/wk x for duration of chemotherapy; I: 70-90% baseline VO2max; D: 60-75mins per week. [50]<br><br>Walking; F: 1 sess/wk x 6wks; I: ns, steps goal	2-3sess/wk x 6wks; I: RPE 13, 1-2sets per exercise; D: ns [90]<br><br>Resistance training using free weights and cable machine (rower, barbell box squats, barbell bench press, barbell conventional deadlift, prone/lying barbell row, cable lat pull down) for 2-4 sets between 6-12 reps; F: 2sess/wk x 12 wks; I: initial loads set at 60% 1 rep max, where load was increased by 1-10kg when individual was able to complete maximum number of reps prescribed; D: 5min warm up + resistance exercise duration (ns) [49]	Walking or cycling and upper and lower limb resistance exercises (ns); F: 3sess/wk x 8wks; I: aerobic 70% of average 6MWT speed, resistance 2-3sets x 10reps initial weights 1.5kg for women and 2kg for men; D:60min [70]<br><br>5 resistance (bicep curl, triceps extension, leg extension, leg curl, abdominal crunch) and aerobic (brisk walking, running indoors or on a treadmill, various cycling activities) training; F:3-5sess/wk x 8wks; I: resistance 2 sets x 8reps 70-75% of 1RM, aerobic 65-75% Hrmax; D:60-75min [73]<br><br>Brisk walking (treadmill or outside) or cycling and 6 resistance exercises (bench press, latissimus pull down, seated row, leg press, leg extension, and leg curl); F:3-4sess/wk x 12 mnths; I: aerobic 60-80% max HR, resistance progressed up to 3 sets x 12 reps (weight progressed if 12 reps >2 sess); D:150min/wk [27]<br><br>Resistance and aerobic exercise training (ns); F: 3sess/wk x 8wks; I: moderate intensity as per ASCM guidelines (ns); D:90min [89]<br><br>Home resistance exercise with free weights and arm ergometer and treadmill walking; F: 5sess/wk x 4wks; I: resistance moderate 3 sets x 10reps 1kg dumbbell and red elastic band, aerobic moderate (ns); D:40min [96]<br><br>Muscle training (muscle strength, muscle endurance and muscle function training) and cardiorespiratory capacity training (eg endurance training) to complete specific number of steps; F: 4sess/wk x 12wks; I: moderate based off RPE (ns); D: 30min [88]<br><br>7 machine-based and body weight resistance exercises (seated cable row, bench press, leg extension, leg press, leg curl, bridge, plank) and aerobic exercises (walking/jogging/running); F: 3sess/wk x 9mnths; I: resistance controlled by max repetition training zones,		extremity stretching, and structured postures and movements to improve balance and strength); F: daily x 8wks; I: ns; D: 45mins [34]<br><br>Yoga (teaching awareness meditation, movement, breathing, and relaxation); F daily x 2wks; I: ns; D: 20mins [36]<br><br>Yoga (meditation, yoga postures, breathing guidance, yoga body scan); F: 1 sess/wk x 8 wks; I: ns; D: 90min [48]

gradually increased by 10% per week; D: ns [43]	aerobic 60-80% Hrmax; D: 70min (resistance 40min, aerobic 30min) [97]<br><br>Aerobic exercise (treadmill walking, jogging, running) and resistance exercise (TheraBand); F: 3sess/wk x 8wks; I: aerobic 60-70% max heart rate, resistance moderate to hard on the Borg Scale; D: aerobic 30mins, resistance 15 mins [46]<br><br>Aerobic exercise (ns) and strength training (ns); F: 2sess/wk x ~4 wks; I: aerobic exercise moderate-intensity, strength training ns; D: 60-90mins (30-45min aerobic exercise, 20mins strength training, 10mins cool down) [41]<br><br>Aerobic exercise (fitness and respiratory exercises, respiratory strengthening exercises, cycle ergometer, Nordic walking, schultz autogenic training) and resistance exercises; F: 5sess/wk x 4 wks; I: Aerobic exercise (only cycle ergometer described) 30-80%peak work rate, resistance exercise at 40-70% resistance exercise; D: aerobic sessions 20-45mins, resistance exercise 20-30mins [35]<br><br>Nordic walking, additional aerobic and resistance exercise; F: 2sess/wk x 26 wks; I: Nordic walking at 55-65% heart rate reserve, other aerobic exercise at 40-75% heart rate reserve, resistance exercise based on 1RM (% ns); D: Nordic walking for 120minutes, aerobic exercise and resistance exercise for 120minutes combined [45]	

Abbreviations: D: duration; F: frequency; HIIT: high intensity interval training; HRmax: heart rate maximum; I: intensity; m: month; min: minutes; mod: moderate; ns: not specified; rep: repetitions; RM: repetition maximum; ROM: range of motion; RPE: rating of perceived exertion; sess: session; wk: week; 6MWT: 6 meter walk test.

Table S3. Overview of all meta-analyses comparing exercise versus non-exercise or usual care. Among the n=71 studies, n=76 intervention arms were evaluated. A negative standardised mean difference (SMD) reflects lower outcome results in the exercise group compared to usual care. A negative SMD favours exercise for pain.

Outcomes and subgroups	No. of study arms	No of participants Exercise vs control	Effect size SMD [95% CI]	Heterogeneity I^2	P value
**Pain (overall effect)**	**76**	**2902 vs 2742**	**-0.45 [-0.62, -0.28]**	**89%**	**<0.001**
Mode					
Aerobic	15	557 vs 542	0.06 [-0.08, 0.20]	18%	0.25
Resistance	15	537 vs 557	-0.38 [-0.61, -0.16]	69%	<0.001
Mixed	25	1048 vs 963	-0.50 [-0.77, -0.23]	88%	<0.001
Other	9	348 vs 330	-1.26 [-2.13, -0.40]	96%	<0.01
Yoga	12	405 vs 354	-0.39 [-0.87, 0.09]	90%	0.11
Length*					
Less than 12 weeks	27	942 vs 892	-0.70 [-1.05, -0.35]	92%	<0.001
12 weeks or longer	42	1662 vs 1552	-0.36 [-0.58, -0.15]	88%	<0.001
Supervision					
Supervised	35	1302 vs 1224	-0.62 [-0.93, -0.31]	93%	<0.001
Unsupervised	41	1600 vs 1518	-0.31 [-0.49, -0.13]	83%	0.001
Pain type as defined by individual Studies**					
Pain (not further specified)	50	2093 vs 2000	-0.51 [-0.71, -0.31]	89%	<0.001
Bodily pain	14	393 vs 347	-0.06 [-0.25, 0.13]	37%	0.53
Musculoskeletal pain	8	202 vs 199	-0.79 [-1.69, 0.10]	94%	0.08
Neuropathic pain	10	444 vs 421	-0.12 [-0.31, 0.07]	47%	0.20
Oral pain	2	50 vs 50	0.05 [-0.60, 0.70]	62%	0.88
Measurement tool**					
Numeric (Pain) Rating Scale	9	304 vs 306	-0.16 [-0.36, 0.04]	28%	0.12
Visual Analog Scale	10	280 vs 276	-0.92 [-1.64, -0.20]	93%	0.01
EORTC QLQ C30 – Pain Scale	26	1120 vs 1039	-0.43 [-0.66, -0.20]	86%	<0.001
SF-36- Bodily Pain Scale	16	411 vs 365	0.07 [-0.21, 0.35]	71%	0.61
Neuropathic Pain scale	3	156 vs 143	-0.23 [-0.78, 0.32]	81%	0.42
Brief Pain Inventory	8	329 vs 295	-0.65 [-1.31, 0.01]	93%	0.05
Western Ontario and McMaster Uni OA Index	2	52 vs 51	0.17 [-0.22, 0.56]	0%	0.39
Cancer type					
Breast	36	1684 vs 1596	-0.54 [-0.78, -0.30]	91%	<0.001
Lung	4	97 vs 86	-1.00 [-2.80, 0.79]	96%	0.27
Prostate	8	278 vs 249	-0.03 [-0.24, 0.18]	29%	0.78
Head and neck	3	96 vs 96	-0.18 [-0.76, 0.40]	74%	0.55
2+ cancer sites	18	532 vs 526	-0.28 [-0.53, -0.03]	74%	0.03
Timing of the intervention***					
During chemotherapy or radiotherapy	39	1719 vs 1652	-0.36 [-0.54, -0.18]	84%	<0.001
After chemotherapy or radiotherapy	19	540 vs 504	-0.60 [-0.98, -0.21]	88%	<0.01
During & after chemotherapy or radiotherapy	5	179 vs 171	0.05 [-0.36, 0.46]	73%	0.81
Sample size					
<20 participants	6	57 vs 56	0.06 [-0.31, 0.43]	0%	0.76
20-59 participants	24	528 vs 471	-0.26 [-0.50, -0.02]	71%	0.03
≥60 participants	46	2262 vs 2145	-0.55 [-0.78, -0.33]	92%	<0.001
Risk of bias					
High risk of bias	37	1287 vs 1248	-0.26 [-0.43, -0.09]	77%	<0.01
Some concerns	27	1033 vs 952	-0.66 [-1.01, -0.32]	92%	<0.001
Low risk of bias	12	570 vs 562	-0.47 [-0.91, -0.03]	92%	0.04
Eligibility criteria					
Pain as inclusion criterion	7	200 vs 183	-0.79 [-1.30, -0.27]	82%	<0.01
Pain not listed as selection criterion	65	2484 vs 2332	-0.45 [-0.64, -0.25]	90%	<0.001
Pain as exclusion criterion	4	136 vs 147	-0.39 [-0.37, -0.04]	42%	0.03

*n=7 intervention arms did not report the exact duration of the exercise intervention. **Multiple data from one study (remembering that five studies include 2 intervention arms) can include one or more of the categories within the above subgroups. ***Treatment stage details during the exercise intervention could not be extracted for all studies.

Table S4. Risk of bias according to RoB 2.

	Randomisation process	Deviations from intended interventions	Missing outcome data	Measurement of the outcome	Selection of the reported result	Overall risk of bias
Bade [40]	+	+	+	!	!	!
Cheville [39]	+	+	+	+	+	+
Galvao [57]	+	+	+	+	+	+
Chuang [53]	+	+	+	+	!	!
Crawford [55]	!	+	+	+	!	!
Klein [61]	+	+	+	+	+	+
Yeo [32]	+	+	!	+	!	!
Dhawan [30]	+	+	+	+	!	!
Backman [52]	!	+	+	+	!	!
Jong [60]	+	+	-	+	!	-
Rogers [64]	+	+	+	+	!	!
Mijwel [62]	+	+	!	+	+	!
Sweeney [66]	+	+	+	+	+	+
Cormie [54]	+	+	+	+	+	+
Eyigor [56]	+	+	-	!	!	-
Cantarero-Villanueva [38]	+	+	+	+	-	-
Galvao [58]	+	+	+	!	+	!
Jiang [59]	+	+	!	+	!	!
Rastogi [63]	+	+	+	!	+	!
Schumacher [65]	+	+	+	!	!	!
van Waart [83]	+	+	+	!	+	!
Wahba [84]	+	+	+	+	+	+
Ammitzboll [29]	+	+	+	+	+	+
Jansen [74]	+	+	+	!	-	-
Cantarero-Villanueva [69]	+	+	+	+	-	-
Prakash [79]	+	+	+	-	-	-
Adeline [28]	!	!	+	-	+	-
Ho [72]	+	+	+	+	+	+
Adams [67]	+	+	+	-	+	-

+ Low risk

! Some concerns

- High risk

Strunk [81]	+	+	!	+	!	!
Nilsen [77]	+	+	+	-	!	-
Vadiraja [82]	+	+	+	-	+	-
Bragante [68]	+	+	+	+	+	+
Hayes [71]	+	+	+	+	+	+
Cavalheri [70]	+	-	+	-	!	-
Porter [78]	+	-	+	-	!	-
Rief [80]	+	-	+	-	!	-
Mijwel [76]	+	-	-	-	!	-
Mijwel [75]	+	-	+	-	!	-
Hojan [73]	+	-	+	-	!	-
Cormie [87]	+	-	+	-	!	-
Wiggenraad [31]	+	-	+	-	!	-
Huberty [92]	+	-	-	-	!	-
Rief [98]	+	-	-	-	!	-
Hammer [91]	!	+	-	-	!	-
Muller [94]	+	-	-	-	!	-
Nyrop [95]	!	-	-	-	!	-
Irwin [27]	!	-	-	-	!	-
Schmidt [99]	!	!	-	-	!	-
Cormie [86]	+	-	-	-	!	-
Hacker [90]	+	-	+	-	!	-
Galiano-Castillo [89]	+	+	!	+	+	!
Park [96]	+	+	+	!	+	!
Dong [88]	+	+	!	+	+	!
Paulo [97]	!	+	+	+	+	!
Bao [85]	!	+	+	+	+	!
Chandwani [100]	!	+	+	+	!	!
Loudon [93]	+	!	+	+	+	!
Knoerl [34]	+	+	+	-	+	-
Heiman [33]	+	+	+	-	+	-
Kuan Yin [46]	+	+	+	!	-	-
Forner [42]	!	+	+	!	+	!
Kang [44]	+	+	+	!	-	-
Sohl [36]	+	+	+	+	-	!
Knoerl [41]	!	+	+	-	-	-
Rutkowski [35]	!	!	-	!	-	-
Rasmussen [49]	+	+	+	+	+	+
Steffens [37]	+	+	+	+	-	-
Sturgeon [50]	+	+	+	-	-	-

Koevoets [45]	+	+	+	-	-	-
Liao [47]	+	+	+	+	!	!
Hacker [43]	+	+	+	!	-	-
Liu [48]	+	+	+	+	!	!
Yang [51]	+	+	+	!	-	-

Table S5. GRADE table of evidence.

Outcome						GRADE assessment						
	K	Study design	N		I^2	Study design	Inconsistency	Risk of Bias	Imprecision	Indirectness	Publication bias	Certainty of evidence
Post exercise intervention vs control group				**Standardised mean difference [95%CI]**								
Pain	71	RCT	5,644	-0.45 [-0.62, -0.28]	89%	Not serious (0)	Not serious (0)	Very serious (-2)[a]	Not serious (0)	Serious (-1)[b]	Not serious (0)	Very low

[a] Evidence was mostly from studies of high risk of bias. [b] Evidence was mostly from studies of unclear risk of indirectnes
